# Assessing anthropogenic impact on the habitat of threatened rock cavy (*Kerodon rupestris*) through its alarm calls

**DOI:** 10.1371/journal.pone.0323711

**Published:** 2025-05-30

**Authors:** Wesley N. Almeida, Kamila S. Barros, Sérgio L. G. Nogueira-Filho, Selene S. C. Nogueira

**Affiliations:** 1 Laboratório de Etologia Aplicada, Universidade Estadual de Santa Cruz, Ilhéus, Bahia, Brazil; 2 Laboratório de Estudo Animal, Universidade do Estado da Bahia, Caetité, Bahia, Brazil; MARE – Marine and Environmental Sciences Centre, PORTUGAL

## Abstract

Acoustic monitoring is emerging as a key tool in wildlife conservation, especially for species in inaccessible habitats like the rock cavy (*Kerodon rupestris*), an endangered species native to Brazil’s threatened Caatinga biome. Emotional stress from threatening situations affects breathing, heart rate, and vocal muscle tension, altering vocal acoustic parameters. This allows researchers to gauge the animal’s environmental perception through its vocalizations. We aimed to evaluate emotional disturbance indicators in free-range rock cavies’ vocalizations to suggest an acoustic index during threats. We compared calls from rock cavies in two areas with similar habitats but that differ in terms of anthropic impacts. Area 1 (A1) is near urban areas and disturbed by livestock and dogs, and Area 2 (A2) is farther from urban areas and free from human disturbance. Data on calls and behaviors were collected *ad libitum* in both areas. The alarm whistle call, making up 73.5% of total calls, was most common. Across 108 observation hours per area, 392 alarm whistle calls were recorded, with more calls in A1 than A2 (223 vs. 169; Chi-square = 29.44, DF = 1, P < 0.001). This resulted in a 32% higher hourly call rate in A1 (2.6 calls/h vs. 1.6 calls/h). Both male and female cavies in A1 had higher high-frequency (F_1, 388_ = 7.80, P = 0.005) and peak-frequency calls (F_1, 388_ = 21.32, P < 0.001). Given the similar landscape and resource availability in both areas, the differences in call emission rate and parameters are likely linked to emotional responses to human disturbances in A1. Thus, alarm whistle calls at an hourly rate of 2.6 calls/h or higher, with high-frequency and peak-frequency at or above 7222 Hz and 2603 Hz, can indicate anthropogenic disturbance in the Caatinga biome, aiding remote monitoring efforts.

## 1. Introduction

The rapid loss of habitats due to anthropogenic causes [1–4] has hastened the decline of Neotropical diversity [5]. To make improvements in the monitoring of free-living animals, studies have explored new technologies such as bioacoustic methods [6–8]. Recent advances in technological and analytical approaches to sounds [9] have allowed researchers to investigate several issues, such as the presence of species [10–13], species richness [14], abundance and density [15].

Acoustic signals have also been used to assess the affective states of wild animals [16–18], both in captivity [19] as well as in a natural wild environment [20,21]. This is possible because when emitting a call an animal uses specific organs such as the larynx, syrinx and vocal fold [16,22], which carry physiological [23], emotional [24,25], and individual information [26,27]. By analyzing call parameters such as frequency, rate, duration, number of notes, and other acoustic features [28], it is possible to assess the perception of the environment as either positive or negative [19], group member recognition [29,30], predator detection [31,32], and the impact of anthropogenic disturbances [33,34].

Alarm calls, for instance, which are reported in several species during threatening situations [35,36], can be widespread in the environment, alerting conspecifics to potential danger [37]. This has suggested acoustic monitoring as a new tool for wildlife conservation [34]. This tool is particularly useful for monitoring species that live in places that are difficult to access. Some studies with the Australasian heron (*Botaurus poiciloptilus*) [12] and African forest elephant (*Loxodonta cyclotis*) [8] are examples of how bioacoustic techniques are effective in monitoring species living in environments where it is extremely difficult to move around and access and collect regular ecological data. These new techniques increase research on data-poor species by providing ecological indicators that can be used for their management and conservation purposes [7,33], such as the rock cavy (*Kerodon rupestris*).

The rock cavy, a rodent from the Caviidae family [38], plays a crucial ecological role in maintaining the diversity of the Caatinga, where it is endemic. The Caatinga, an exclusively Brazilian biome, is currently under threat [39], and *Kerodon rupestris* is at the base of the food chain of the predators that occur in this region, such as the jaguar (*Panthera onca*), cougar (*Puma concolor*), ocelot (*Leopardus pardalis*), wild fox (*Cerdocyon thirty*), oncilla (*Leopardus tigrinus*) and laughing falcon (*Herpetotheres cachinnans*) [40–42]. Additionally, the species is also used as an alternative source of animal protein by the human populations that inhabit the Caatinga and live below the poverty line [43–45]. Nevertheless, the rock cavy is threatened due to the accelerated process of environmental change and deterioration caused by the unsustainable use of natural resources. This has led to the rapid loss of this endemic species [46]. Due to a 30% population decline over the last 10 years, the species has been included in the vulnerable category [47]. However, monitoring activities to protect the species and its habitat in the area are challenging due to the conditions of the Caatinga. The Caatinga region has a complex topography, consisting of a rocky landscape, which makes it challenging to move around. Additionally, only 7% of the original Caatinga area is protected by conservation units [39]. Environmental stressors caused by human activities, such as habitat degradation, can threaten biodiversity and can also cause suffering to individuals, impacting their welfare [see 48]. Thus, stressor agents affect animals’ acoustic behavior [16,21,48]. It is therefore urgent to conduct studies that identify ecological indicators using non-invasive techniques such as bioacoustics for assessing the rock cavy’s response to environmental disturbances using remote acoustic monitoring.

The species lives in a harem, usually composed of one male and three to four adult females and their offspring [49]. Adapted to a semi-arid environment, it lives in rocky terrain, using rock crevices as shelter [50]. The acoustic repertoire of *K. rupestris* comprises 11 different types of calls and two mechanical signals [49,51]. Among them, the whistle is an alarm call emitted when the animal is facing threatening situations, such as the presence of a potential predator or during intraspecific agonistic interactions [49,51,52]. The alarm whistle call is thus related to a negative affective state caused by negative events such as predator attack or aggression between conspecifics, both associated with high arousal (e.g., to run). Therefore, we aimed to compare the type and the number of calls, in addition to the acoustic parameters of alarm whistle calls emitted by *K. rupestris* living in two areas with different degrees of anthropic impact. We expected a higher emission of alarm calls in the area with greater anthropogenic disturbance than in the less disturbed one. Additionally, if high-pitched calls are emitted in hostile contexts [16,53] and are related to negative motivational states in animals [54,55], we also predicted that the calls emitted by rock cavies in the more anthropogenically disturbed area would exhibit higher frequencies compared to those from the less disturbed area.

## 2. Materials and methods

### 2.1. Ethics statement

This study was approved by the Chico Mendes Institute for Biodiversity and Conservation (ICMBio/SISBIO) (#59323), the Environment and Water Resources Institute of Bahia (INEMA) (#20065), and the Commission for Ethics in the Use of Animals (CEUA) of the State University of Bahia (UNEB) (Protocol #07/2019).

### 2.2. Study area and animals

The study was carried out with free-ranging animals in two interconnected conservation units (14°16’ 14°39’S 42°46’ 43°46’W): Serra dos Montes Altos wildlife refuge (REVIS) and Serra dos Montes Altos state park (PESMA), both covering approximately 46,000 Ha. These conservation units are located in the southwestern part of Bahia state, Brazil, and are monitored by the regional environmental agency. Both REVIS and PESMA are characterized by a semi-arid climate, an ecotone transition area between herbaceous and arboreal caatinga vegetation, rupestrian fields and gallery forests [56]. Topographically, both conservation units are characterized by the São Francisco River basin depression and a sedimentary structural plateau – the *Espinhaço* Plateau [57]. There are several endemic and rare species living in these areas, highlighting the importance of maintaining this habitat [58]. These conservation units contain two distinct sampling areas, named Serra dos Montes Altos wildlife refuge (A1) and Serra dos Montes Altos state park (A2), located within 15 km of each other.

Both areas (A1 and A2) share similar natural landscapes. They are characterized by large rock formations with dystrophic litholic neosol and dystrophic red-yellow latosol pedology [59], proximity to springs and bodies of water, and a predominance of shrubby and herbaceous vegetation strata typical of the Caatinga, composed of dry branches and thorny plants, with overall vegetation cover classified as savanna formation [60]. They present similar hypsometric data, ranging from 750 m to 1,150 m [61], and show no signs of recent wildfires or other severe anthropogenic alterations in the landscape. The availability of water and shelter is also similar. However, A1 (86,675.03 m², 14°19’07” S 43°03’42” W) is located inside REVIS and was characterized as the most disturbed area due to its hunting history. Additionally, this area is 630 m away from the urban zone, and shows anthropogenic changes, such as drinking troughs arranged along the trails for domestic animals that usually pass by. There are fences delimiting the area for animals, the presence of feral and domestic dogs, small livestock production, farmers, and a pipe network for water distribution. In contrast, A2 (401,212.48 m²; 14°25’45” S 42°57’51” W) was characterized as the less disturbed area, mainly because it receives few visits due to difficult access. This area is located inside PESMA and 6.1 km away from the nearest urban area. In this area there are no drinking troughs, fences, livestock, domestic or feral dogs, characterized as the opposite of A1.

The sample areas were chosen during the 10-day pilot period that occurred prior to data collection. During this period, we conducted indirect searches for animal traces, such as footprints and feces, and registered general activities and behaviors of animals (see Table 1). The rock cavy has latrine behavior, which makes its detection easier in the wild [50]. Moreover, the park rangers, who monitor the security of these sites and usually sight the rock cavy groups, helped to determine the sample areas. During data collection we recorded quite a similar number of rock cavies in both areas (A1 = 91 and A2 = 97).

**Table 1:** Description of the emission context and behavioral category of different call types emitted by rock cavies according to the age (A: adults and J: juveniles) and sex (M: male, F: female and U: Unidentified) of the caller.

Call	Age and sex	Behavioral category	Context
Alarm Whistle	A, F, M	Alarm Agonistic	Call emitted in a threatening situation, when the individual is surprised by some disturbance, such as the approach of humans or other possible predators (medium-sized carnivorous mammals). The animal assumes an alert posture, standing on a rock (usually the tallest) or on the ground, with its body tilted at 90°, head forward, and vocalizes. Call emitted during conflict between conspecifics. The caller stands still in a coping posture and vocalizes until the aggression begins.
Snort-like	A, F	Agonistic	Call emitted prior to a conspecific attack onslaught. The caller stands still, with the head slightly directed towards the aggressor, and vocalizes until being attacked and expelled from the rock crevice.
Snort	A, F	Agonistic	Call emitted in association with snort-like vocalization. The caller assumes a submissive posture and vocalizes until being attacked and expelled from the rock crevice by the aggressor.
Scream	J, U	Social isolation	Call emitted by an individual isolated from the group. The caller stands still with its head slightly raised above its body and moves it from side to side and vocalizes.
Whine	J, U	Pain	Call emitted by an individual after being attacked by an interspecific (feral dog). After being captured and bitten, the caller remained lying down, with the head directed to the interspecific and vocalizing.

### 2.3. Data collection

The study was carried out from November 2019 to March 2020, with a total of 24 expeditions (A1: 12 A2: 12). We interrupted our data collection in March 2020 due to the COVID-19 pandemic, when all conservation units and national reserves in Brazil were closed to visitors. All acoustic calls and associated behaviors were collected simultaneously (Table 1) by *ad libitum* method [62]. One observer (W. N. A.) and a field assistant monitored and registered the emissions of animals’ calls and described the associated behaviors. During the field period, the expeditions were interspersed between the two areas, two days in A1 and two days in A2 and so on. Given that there are conflicting findings regarding the species’ peak activity period [48,63–65], the observation sessions took place between 6h00 and 17h00 (encompassing the crepuscular hours of the early morning), totaling 216 hours of data collection (A1: 108 h and A2: 108 h). The observation session was chosen following reports on the species’ general activity [49,66] and confirmed here during the pilot period.

We used a portable digital Tascam DR-44WL recorder (Tokyo, Japan), coupled to a Sennheiser ME-66 unidirectional microphone (Wedemark, Germany) to record the emitted calls. The recorder settings were mono mode, WAV format, 48 kHz sampling rate and 16-bit resolution. To capture the beginning of the sound emission of the animals, we used the “pre-REC” function programmed in 5s. The observer manually activated the recorder as soon as the individuals emitted a call, without cuts until the call ended.

The observation sites had been previously selected during the pilot period, when the observer chose the locations that provided the best view of the animals. From these sites, we first habituated the animals to the presence of the observer to minimize the observation impact that occurred during the pilot period. During data collection, the observer used trees as observation sites, keeping a distance of approximately 2.0 m to 2.5 m from the animals. There were eight observation sites in A1 and nine observation sites in A2. The observer occupied these observation sites and set up the equipment at least 40 min before the animals returned to their general activities outside their burrows. The identification of the sex of the animal was made from direct visualization of the animal’s genital tract or, if in doubt, the observer recorded the animal as unidentified sex category (Table 2).

**Table 2:** Number of call types emitted by rock cavies according to the age (adult and juvenile) and sex (female, male and unidentified) of the caller and the area of occurrence (A1: most disturbed area; A2: less disturbed area) in the Brazilian Caatinga.

Vocalization	Female (Adult)		Male (Adult)		Unidentified		Juvenile	
	A1	A2	A1	A2	A1	A2	A1	A2
Alarm whistle	124	138	99	31	54	7		
Snort-like	77	48						
Snort	10	5						
Scream							12	
Whine							11	

### 2.4. Acoustic analysis

The call emissions of *K. rupestris* were classified by aural and visual inspection of spectrograms using Raven Pro Software version 1.6 (Cornell Lab of Ornithology, Ithaca, NY). The following settings were used: Hann type window, window size 1351 samples, 90% overlap in time domain, and DFT size 4096. The smallest vocal unit of calls was called a pulse, but it can also be called an element, note or syllable, defined as a continuous sound without internal interruptions, following Barros et al. [29]. For each pulse (element), the following parameters were measured: high-frequency (Hz) (higher frequency threshold), low-frequency (Hz) (lower frequency threshold), peak-frequency (Hz) (frequency that corresponds to the maximum occurrence of power in the selection of elements or phrases), and duration (total duration in seconds) [67]. The emission rate per hour of each call or hourly rate (HR) was calculated by dividing the total number of each call type by the total observation time for each sampled area.

### 2.5. Data and statistical analysis

First, we described the calls and their associated behaviors to better understand their possible functions (see Table 1). We then quantified the number of call types emitted by rock cavies according to age, caller’s sex and area of occurrence (Table 2). The age classification was based on the observation of individual size, as adults and juveniles are distinguishable by body size [68]. We compared the emission of the different calls according to the sampled area (A1 and A2) using the chi-square test. Following that, we used discriminant function analysis (DFA) [69] of the acoustic parameters (low-frequency, high-frequency, peak-frequency, and duration) to test the classification of the different calls initially categorized by aural and visual inspection. Prior to conducting the DFA analysis, we normalized and standardized the variables by subtracting the mean of each variable from its corresponding data points and dividing the result by the variable’s standard deviation, a process known as Z-score transformation. This standardization was necessary to prevent the spurious attribution of weights to acoustic parameters measured in different units. Thereafter, we performed the DFA cross-validation test to ascertain whether the prediction for each vocal type based on the measured acoustic parameters was correct.

Snort-like and snort calls were emitted exclusively by adult females, while scream and whine calls did not occur very often and were only emitted by juvenile animals in the most disturbed area (A1) (Table 2). In turn, the alarm whistle was the prevalent call, emitted exclusively by adult rock cavies within both sampled areas (Table 2). Therefore, we applied general linear mixed models (GLMs) to verify whether the high-frequency as well as other acoustic parameters (low-frequency, peak-frequency, and duration) varied according to the sampled areas (A1 and A2) just for alarm whistle calls. We applied one model per acoustic parameter (high-frequency, low-frequency, peak-frequency, and duration). In these models we included the sex (male and female), the sampled areas (A1 and A2), and the interaction between them (sex x area) as fixed factors. When the interaction was significant, we used Tukey HSD *post hoc* tests. We graphically checked the residuals of every model for normal distribution and homoscedasticity, and all data were Box-Cox transformed to meet these assumptions. For all analysis, we used the MINITAB 19.0 program (Minitab Inc.) considering α < 0.05.

## 3. Results

### 3.1. Recordings and discrimination of calls

**Fig 1:**
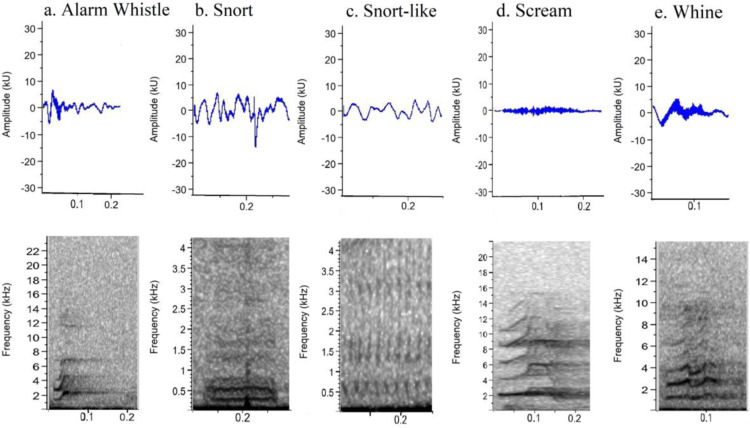
Oscillograms and spectrograms of the five calls emitted by rock cavies in two areas of the Brazilian Caatinga.

We recorded a total of 616 calls (A1: 387; A2: 229), which were initially aural and visual, inspected the generated spectrograms and classified them into five types of calls (Fig 1, see also S1–S5 Files). *K. rupestris* emitted these five types of calls while performing four behavioral patterns: alarm, agonistic, social isolation, and pain (Table 1).

**Fig 2:**
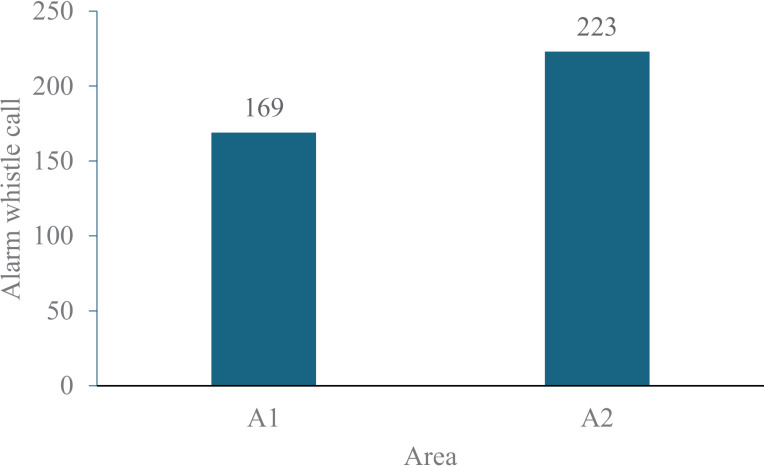
Number of alarm whistle calls emitted by rock cavies in two areas of Brazilian Caatinga.

The alarm whistle calls accounted for 73.5% and were emitted by adult animals of both sexes in both areas (Table 2). There were, however, a greater number of alarm whistle calls emitted in the more disturbed area (A1) than in the less disturbed area (A2) (Chi-square = 29.44, DF = 1, *P* < 0.001, Fig 2). However, this disparity between the two areas was entirely attributable to males (Chi-square = 35.57, DF = 1, *P* < 0.001 and not to females (Chi-square = 0.75, DF = 1, *P* = 0.387). The total observation time was the same (108 hours) for both sampled areas, which led to an hourly rate (HR) for the alarm whistle call that was 32% higher in A1 than A2 (2.6 calls/h *vs* 1.6 call/h, respectively).

The second most prevalent call was the snort-like call (20.2%) (A1: HR = 0.7 call/h A2: HR = 0.4 call/h). This call was more frequently emitted in the more disturbed area (A1) compared to the less disturbed area (A2) (Chi-square = 6.73, DF = 1, *P* = 0.009). The third most prevalent call was the snort call (2.4%) (A1: HR = 0.09 call/h A2: HR = 0.04 call/h) and was similarly emitted in both areas (Chi-square = 1.67, DF = 1, *P* = 0.197). The snort-like call and the snort call were emitted only by females across the two areas (Table 2). We recorded screams (1.9%) and whines (1.8%) only in the most disturbed area (scream HR = 0.1 call/h and whine HR = 0.1 call/h). Additionally, these last calls were emitted only by juveniles (Table 2).

Feral dogs were observed exclusively in the most disturbed area (A1). During the 12 expeditions conducted in A1, feral dogs were seen near or around the data collection points and roaming the access trails. These dogs posed a threat to *K. rupestris*, as we witnessed a *Canis lupus familiaris* catching and killing a rock cavy. In response to the presence of feral dogs, six alarm whistle calls were emitted. Five more alarm whistle calls were emitted in response to humans walking through A1. Additionally, one scream call was recorded due to the presence of feral dogs nearby. During the collection period in A1, two to three dogs were present in each of the alarm whistle call recordings where a dog threat was detected. No natural predators were observed, and there was no evidence of their presence in A1. No dogs or humans were seen in in the less disturbed area (A2). However, during the observation, we detected a direct threat from the natural predator *Eira barbara*, which triggered the emission of two alarm whistle calls in A2.

**Fig 3:**
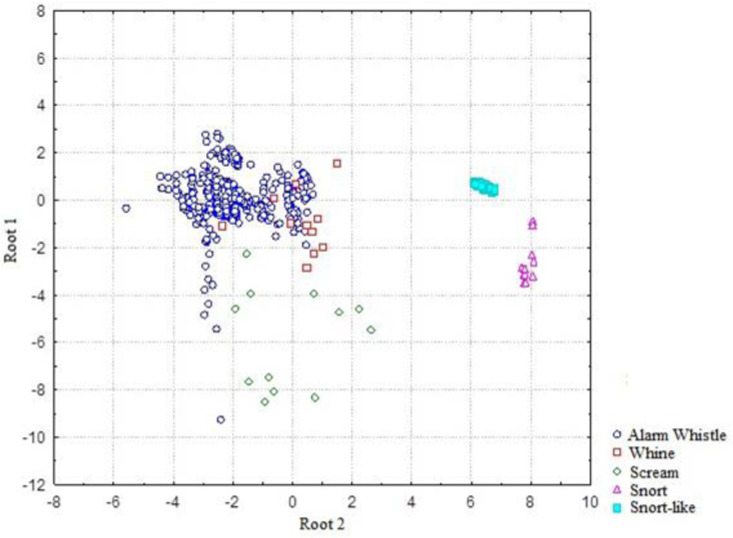
Graphic representation of the dispersion of the individual scores of the first and second canonical roots resulting from a discriminant analysis using four acoustic parameters (minimum frequency, maximum frequency, duration, and dominant frequency) of the alarm whistle (N = 453), snort-like (N = 125) snort (N = 15), whine (N = 12) and scream (N = 11) emitted by rock cavies (N = 26) recorded in two areas of the Brazilian Caatinga.

The DFA of all calls showed differences among the five call types (Wilks λ = 0.03, F_16, 1858_ = 245.91, P < 0.0001, DFA = 0.86, Table 3), confirming and validating the initial inspection, with a cross-validation value of 0.86 (see raw data in S6 File). All acoustic parameters used for DFA contributed to differentiating the types of calls signaled (Fig 3).

**Table 3:** Means (±standard errors) of acoustic parameters and the classification through discriminant function analysis (DFA) of different types of calls (N = 616) emitted by rock cavies recorded in the Brazilian Caatinga.

Acoustic parameter	Alarm Whistle	Snort-like	Snort	Scream	Whine
High-frequency (Hz)	8434.17 (±156.38)	6170.92 (±3.98)	856.06 (±5.90)	14932.60 (±4310.67)	10512.58 (±961.37)
Low-frequency (Hz)	1201.02 (±6.11)	258.40 (±3.52)	157.32 (±4.89)	913.80 (±55.04)	920.53 (±29.81)
Peak-frequency (Hz)	2508.27 (±359.37)	5362.62 (±158.86)	305.6 (±160.65)	2347.11 (±251.45)	19301.58 (±211.84)
Duration (s)	0.23 (±0.01)	0.02 (±0.02)	0.27 (±0.01)	0.55 (±0.04)	0.23 (±0.02)
Total (N)	453	125	15	12	11
DFA	0.82	1.00	1.00	0.92	0.91
Cross-validation	0.82	1.00	1.00	0.92	0.91

### 3.2. Acoustic characteristics of alarm whistle calls according to the sampled areas

**Fig 4:**
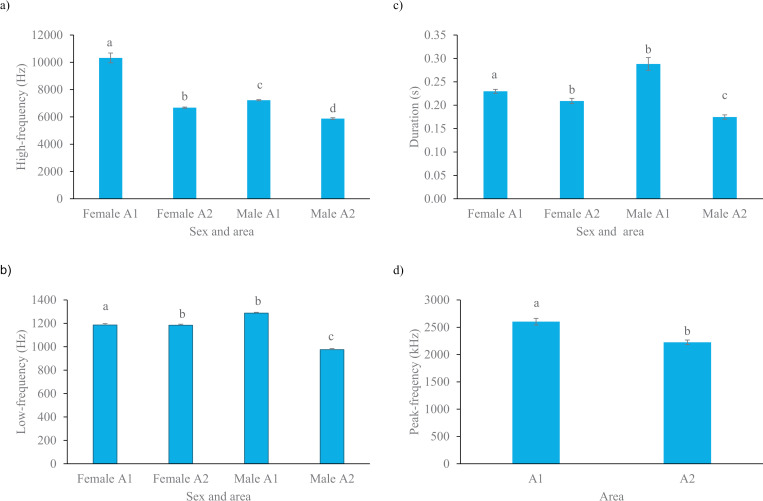
Means (±standard error) of the high-frequency (a), low-frequency (b), peak-frequency (c), and duration (d) of alarm whistle calls emitted by rock cavies in the Brazilian Caatinga according to the caller’s sex and/or area of occurrence (A1: most disturbed area; A2: less disturbed area). Columns that do not share the same letter differed according to Tukey’s post hoc tests (*P* < 0.05).

The interaction between the sex and area affected the high-frequency type of alarm whistle call (F_1, 388_ = 7.80, *P* = 0.005). The *post hoc* tests showed that both female and male rock cavies living in the most disturbed area (A1) emitted alarm whistles with a higher high-frequency than those living in the less disturbed area (A2). This difference was found to be 1.3 times more pronounced in females than in males (Fig 4a). The interaction between the sex and area also affected the low-frequency (F_1, 388_ = 148.52, *P* < 0.001) and the duration of the alarm whistle calls (F_1, 388_ = 27.14, *P* < 0.001). The *post hoc* tests showed that low-frequency calls (Fig 4b) and duration (Fig 4c) only differed in males between both areas (males in A1 emitted longer whistle calls with higher low frequency), than females living in the same area and both females and males living in the less disturbed area (A2). The peak-frequency (F_1, 388_ = 21.32, *P* < 0.001) of the rock cavy’s alarm whistle calls was only affected by the sample areas. Rock cavies living in the most disturbed area (A1) emitted alarm whistles with a higher peak-frequency than those living in the less disturbed area (A2) (Fig 4d) (see raw data and complete statistical analysis in – S7 File).

## 4. Discussion

Our results showed five types of calls emitted by free-ranging *K. rupestris*, which was a smaller acoustic repertoire than that reported for captive animals (Lacher [49]: six types of calls; Monticelli and Alencar-Junior [51]: 11 types of calls and two mechanical signals). Different stimuli in the two environmental conditions (wild x captive) and the proximity to the caller may explain the different number of types of calls reported in these studies.

The snort-like and snort calls recorded in both sampled areas were emitted exclusively by adult females and are not easy to record in the wild. They are barely perceptible to the human, and only at a close distance (within 3 m from the caller). The snort call described by Monticelli and Alencar-Junior [51] seems to be the same call recorded in the present study, as they occurred in a similar agonistic context. Alencar-Junior [52] described an association between alarm whistle and snort calls occurring in a territorial dispute, while the caller was under attack. This description is compatible with the behavioral context described in the present study for both the snort and snort-like calls, when only the individual who is attacked and expelled from the rock site emits these calls. Thus, the emission of snort and snort-like is probably related to a submissive behavior. This is compatible with a hierarchically organized species, such as *K. rupestris* [49]. This kind of vocal subordination signal has also been described for other mammalian species (grunt calls in *Papio cynocephalus* [70]; giggle calls in *Crocuta crocuta*, [71]; grunt calls in *Tayassu pecari* [72]). However, further experimental study must be done to test this hypothesis for *K. rupestris*. Our study draws heavily on previous foundational work about the repertoire of this species [51]. However, it also diverged by introducing new methodologies and findings that enrich our understanding of the rock cavy’s acoustic behavior, diversity, and plasticity. For instance, our study reveals that males and females adjust their vocalizations differently, and males in disturbed areas exhibit multidimensional vocal modulation (details below). Moreover, the snort-like call is not comparable to any call described before [49,51]. Therefore, this would be a new call to be included in the species’ acoustic repertoire. This underscores the importance of continued investigation to fully capture the intricate vocalizations of this species.

The scream and whine calls did not occur very often and were only emitted by juvenile animals in the most disturbed area (A1). The scream was categorized herein as an emission of pain, emitted when an individual was captured and bitten by a feral dog (*Canis lupus familiaris*). It is reasonable to suppose that the scream is the squeal call, previously described by Lacher [49] for both the rock cavy and the guinea pig (*Cavia aperea*). The author describes the squeal call in the context of fear and pain, during handling of captive animals and after being bitten by a conspecific [49]. In our study, the whine was emitted by an individual isolated from the group; a rare context for free-ranging rock cavy due to their cohesive nature. The spectral features of whine and scream described in the present study were like the whine and yelp calls described before for the rock cavy [51], which indicates the similarity of the calls emitted both in wild and captivity conditions. However, screams and whines are underrepresented, with only 12 and 11 samples, respectively. This limited data collection may affect the robustness of conclusions drawn about these less frequent vocalizations. Therefore, further studies should aim to obtain larger datasets to better understand the occurrence and functions of these calls, especially in different environmental contexts and age groups.

The alarm whistle was the most prevalent call emitted by the rock cavies in both areas. The emission of this call occurs in contexts of alarm and aggression and only by adult individuals, as previously reported [49,51]. Therefore, it is reasonable to suppose that the alarm whistle can exert both functions, acting as an alarm call after predator detection and for disputes between conspecifics, as described for other mammals [73]. Further studies must be done to clarify the function of this call, as already done for calls of other caviomorphs (e.g., *Hydrochoerus hydrochaeris* [30,74]).

The overall higher emission of high-pitched alarm whistle calls is associated with the presence of humans and negative anthropic changes in the environments [21,75–77]. Humans and exotic predators (domestic and feral dogs) that usually transit in A1 may be the main stimuli for the high rate of emission of alarm whistle calls and changes in their acoustic parameters. During the data collection period, no exotic predators were observed, and no humans other than the observer and the field assistant were present in the less disturbed area (A2). Anthropogenic actions usually enforce new challenges for free-ranging animals [78–80]. In these disturbed contexts, animals can respond differently to ensure survival and reproductive success, which can generate new selective pressures [21,54,81]. Alarm calls are usually emitted by threatened animals [35], which need to balance the cost and benefit for their emission [82,83]. These calls are louder and can easily expose the caller to a nearby audience, turning it into easy prey for opportunistic predators [37], as described for the European pied flycatcher (*Ficedula hypoleuca*) [84] and vervet monkey (*Chlorocebus pygerythrus*) [85].

Our results suggest that the interaction between sex and area has influenced the acoustic characteristics of alarm calls in *K. rupestris*, implying that males and females adjust their vocalizations differently in response to environmental disturbance. Both males and females in the more disturbed area (A1) emitted alarm calls with higher high-frequencies compared to individuals in the less disturbed area (A2). However, only males from A1 produced calls with higher low-frequencies and longer durations, highlighting a multidimensional vocal modulation in males. This pattern may be related to differences in social and ecological roles between the sexes. Males of *K. rupestris*, being more territorial and frequently involved in agonistic interactions [49,52], may exhibit greater vocal flexibility to ensure efficient detection of their calls by both group members and potential competitors. The emission of longer calls with greater frequency variation may enhance signal propagation in a fragmented environment, thereby maximizing their alert function [86,87]. In contrast, females, by adjusting only the high frequency, may be reducing predator exposure and minimizing the energy costs associated with vocalization [28]. These findings seem to show vocal plasticity in *K. rupestris*.

It is important to highlight the limitation of our study due to the absence of replicates for each level of disturbance (A1 disturbed area; A2 undisturbed area); due to this, other factors could also play a role, besides the anthropogenic factors such as diseases, parasites, and social events, for instance. However, given the similarity in habitat structure and other variables, such as temperature and vegetation cover, as well as food, water, and shelter resources for rock cavies in both areas, it is reasonable to infer that those variations in the emission rate and acoustic parameters of rock cavy whistle calls between areas may be linked to the animals’ emotional state in response to human disturbance in the most disturbed area. Therefore, the emission of alarm whistle calls with an hourly rate of 2.6 calls/h or higher, along with high-frequency and peak-frequency of 7222 Hz and 2603 Hz or higher, respectively, may serve as ecological indicators of anthropogenic disturbance in the Caatinga biome, enabling remote monitoring. Furthermore, future studies focusing on monitoring animals’ distress in response to negative events—such as by measuring cortisol levels and correlating these with acoustic variations—could provide valuable insights into the relationship between whistle call emissions, negative emotional states, and high arousal [53,54]. This approach could enhance our understanding of the stressors influencing vocalizations and offer a more comprehensive ecological indicator of anthropogenic impact.

In addition, the *ad libitum* method of vocalization collection used herein played a crucial role in documenting vocalizations within our study. This method provided the flexibility needed to capture a wide range of vocal behaviors in a natural setting, reflecting the genuine conditions of the animals’ environment. However, while *ad libitum* sampling allows for comprehensive data collection without strict constraints, it is important to acknowledge its limitations. Systematic recording methods, on the other hand, offer the advantage of yielding more precise insights into specific phenomena, such as predator presence or isolation frequency. These methods involve structured and consistent data collection protocols, which can enhance the reliability and comparability of the findings. Thus, future research directions could explore a comparison of these approaches. Therefore, conducting parallel studies using both *ad libitum* and systematic recording methods could provide a deeper understanding of their respective strengths and limitations. This comparative analysis would help identify the most effective strategies for documenting vocalizations and interpreting their ecological significance.

As previously stated, *K. rupestris* and its habitat are under threat. However, the conservation units in the Caatinga are not adequately protected due to insufficient financial and human resources. For instance, only six park rangers are responsible for enforcing the law across the entire 46,000 hectares of the two conservation units studied, namely the Serra dos Montes Altos wildlife refuge (REVIS) and the Serra dos Montes Altos state park (PESMA). Utilizing the results of this study will enable remote bioacoustics monitoring, allowing authorities to better allocate scarce resources to areas with higher levels of human interference. Nonetheless, due to the study’s limitations, such as the lack of behavioral quantification data in the observed areas, further research is required to validate this hypothesis. Moreover, additional study may be required to determine whether *K. rupestris* adjusts its acoustic responses based on the type of threat, which would improve remote monitoring. Furthermore, long-term monitoring could be suggested to assess the stability of observed patterns and their potential changes over time in response to varying anthropic pressures.

## 5. Conclusions

The acoustic characteristics of alarm whistle calls emitted by *K. rupestris* in the most disturbed area can be attributed to negative events and high arousal. Although our study had limitations that prevented more systematic tests on the emotions associated with negative events experienced by *K. rupestris*, our findings allow us to infer the quality of their environment and, consequently, the rock cavy’s vocal responses, suggesting impaired animal welfare in the most disturbed area. These results validate our predictions and highlight the potential of using acoustic monitoring of alarm whistle calls by free-ranging *K. rupestris* as an ecological indicator of environmental disturbance in the Caatinga biome.

## Supporting information

S1 File Example of rock cavies’ alarm whistle recorded in the Caatinga. (WAV)

S2 File Example of rock cavies’ scream recorded in the Caatinga. (WAV)

S3 File Example of rock cavies’ snort recorded in the Caatinga. (WAV)

S4 File Example of rock cavies’ snot-like recorded in the Caatinga. (WAV)

S5 File Example of rock cavies’ whine recorded in the Caatinga. (WAV)

S6 File Raw data and DFA analysis of rock cavies’ calls recorded in the Caatinga. (DOCX)

S7 File Raw data and generalized linear models (GLMs) analyses of rock cavies’ alarm whistle calls recorded in the Caatinga. (DOCX)
