# Supplementary material for: Assessing anthropogenic impact on the habitat of threatened rock cavy (Kerodon rupestris) through its alarm calls
Source: PLoS One. 2025 May 30;20(5):e0323711. doi: 10.1371/journal.pone.0323711 (PMC12124852; doi:10.1371/journal.pone.0323711)
Supplement: S6 File — (DOCX) [file pone.0323711.s006.docx]

| Discriminant Analysis: Type versus Low.Freq; High.Freq; Delta time; Peak.Freq | | | | | | |
| --- | --- | --- | --- | --- | --- | --- |
| Linear Method for Response: Type | | | |  |  |  |
| Predictors: Low.Freq; High.Freq; Delta time; Peak.Freq | | | | | |  |
| Groups |  |  |  |  |  |  |
|  |  |  |  |  |  |  |
| Group | Alarm whistle | Whine | Scream | Snort | Snort-like |  |
| Count | 453 | 11 | 12 | 15 | 125 |  |
| Summary of Classification | | |  |  |  |  |
|  |  |  |  |  |  |  |
|  |  |  |  |  |  |  |
| True Group | |  |  |  |  |  |
| Put into Group | Alarm whistle | Whine | Scream | Snort | Snort-like |  |
| Alarm whistle | 370 | 1 | 0 | 0 | 0 |  |
| Whine | 79 | 10 | 1 | 0 | 0 |  |
| Scream | 4 | 0 | 11 | 0 | 0 |  |
| Snort | 0 | 0 | 0 | 15 | 0 |  |
| Snort-like | 0 | 0 | 0 | 0 | 125 |  |
| Total N | 453 | 11 | 12 | 15 | 125 |  |
| N correct | 370 | 10 | 11 | 15 | 125 |  |
| Proportion | 0.82 | 0.91 | 0.92 | 1.00 | 1.00 |  |
| Correct Classifications | | |  |  |  |  |
|  |  |  |  |  |  |  |
| N | Correct | Proportion | |  |  |  |
| 616 | 531 | 0.862 |  |  |  |  |
|  |  |  |  |  |  |  |
| Summary of Classification with Cross-validation | | | | |  |  |
|  |  |  |  |  |  |  |
| True Group | |  |  |  |  |  |
| Put into Group | Alarm whistle | Whine | Scream | Snort | Snort-like |  |
| Alarm whistle | 366 | 1 | 0 | 0 | 0 |  |
| Whine | 83 | 10 | 1 | 0 | 0 |  |
| Scream | 4 | 0 | 11 | 0 | 0 |  |
| Snort | 0 | 0 | 0 | 15 | 0 |  |
| Snort-like | 0 | 0 | 0 | 0 | 125 |  |
| Total N | 453 | 11 | 12 | 15 | 125 |  |
| N correct | 366 | 10 | 11 | 15 | 125 |  |
| Proportion | 0.81 | 0.91 | 0.92 | 1.00 | 1.00 | Cross Validation |
| Correct Classifications with Cross-validation | | | | |  |  |
|  |  |  |  |  |  |  |
| N | Correct | Proportion | |  |  |  |
| 616 | 527 | 0.856 |  |  |  |  |
| Squared Distance Between Groups | | | |  |  |  |
|  |  |  |  |  |  |  |
| Put into Group | Alarm whistle | Whine | Scream | Snort | Snort-like |  |
| Alarm whistle | 0 | 7.192 | 41.176 | 111.897 | 72.357 |  |
| Whine | 7.192 | 0 | 24.342 | 70.548 | 40.7 |  |
| Scream | 41.176 | 24.342 | 0 | 84.614 | 83.92 |  |
| Snort | 111.897 | 70.548 | 84.614 | 0 | 18.973 |  |
| Snort-like | 72.357 | 40.7 | 83.92 | 18.973 | 0 |  |
| Linear Discriminant Function for Groups | | | |  |  |  |
|  |  |  |  |  |  |  |
|  | Alarm whistle | Whine | Scream | Snort | Snort-like |  |
| Constant | -65.691 | -45.085 | -69.324 | -7.233 | -5.837 |  |
| Low.Freq | 0.095 | 0.074 | 0.065 | 0.005 | 0.024 |  |
| High.Freq | 0.002 | 0.002 | 0.003 | 0 | 0.001 |  |
| Delta time | 0.797 | 14.149 | 81.331 | 50.371 | -3.389 |  |
| Peak.Freq | 0 | 0 | 0 | 0 | 0 |  |
