# Supplementary material for: Assessing anthropogenic impact on the habitat of threatened rock cavy (Kerodon rupestris) through its alarm calls
Source: PLoS One. 2025 May 30;20(5):e0323711. doi: 10.1371/journal.pone.0323711 (PMC12124852; doi:10.1371/journal.pone.0323711)
Supplement: S7 File — (DOCX) [file pone.0323711.s007.docx]

GLMs whistle calls’ acoustic parameters

| Worksheet 1 | |  | |  | |  |  | | | |  |  |
| --- | --- | --- | --- | --- | --- | --- | --- | --- | --- | --- | --- | --- |
| General Linear Model: Duration versus Sex, Area | | | | | | | | | | |  |  |
| **Method** | |  | |  | |  |  | | | |  |  |
| Factor coding | (-1, 0, +1) |  | |  | |  |  | | | |  |  |
|  |  |  | |  | |  |  | | | |  |  |
| Box-Cox transformation | |  | |  | |  |  | | | |  |  |
| Rounded λ | 0 |  | |  | |  |  | | | |  |  |
| Estimated λ | 0.108971 |  | |  | |  |  | | | |  |  |
| 95% CI for λ | (-0.0705295, 0.281471) | | |  | |  |  | | | |  |  |
| **Factor Information** | | | |  | |  |  | | | |  |  |
| **Factor** | **Type** | **Levels** | | **Values** | |  |  | | | |  |  |
| Sex | Fixed | 2 | | F, M | |  |  | | | |  |  |
| Area | Fixed | 2 | | A1, A2 | |  |  | | | |  |  |
| **Analysis of Variance for Transformed Response** | | | | | | | | | | |  |  |
| **Source** | **DF** | **Adj SS** | | **Adj MS** | | **F-Value** | **P-Value** | | | |  |  |
| Sex | 1 | 0.0391 | | 0.03909 | | 0.37 | 0.543 | | | |  |  |
| Area | 1 | 6.136 | | 6.13597 | | 58.31 | 0 | | | |  |  |
| Sex*Area | 1 | 2.8559 | | 2.85587 | | 27.14 | 0 | | | |  |  |
| Error | 388 | 40.8271 | | 0.10522 | |  |  | | | |  |  |
| Total | 391 | 49.4657 | |  | |  |  | | | |  |  |
| **Grouping Information Using the Tukey Method and 95% Confidence** | | | | | | | | | | | | |
| **Area** | | | **N** | | **Mean** | | | **Grouping** | |  | |  |
| A2 | | | 223 | | 0.257294 | | | A |  | 392 | |  |
| A1 | | | 169 | | 0.191098 | | |  | B |  | |  |
| Means that do not share a letter are significantly different. | | | | | | | | | | | |  |
| **Tukey Pairwise Comparisons: Sex*Area** | | | | | | | | | |  | |  |
| **Grouping Information Using the Tukey Method and 95% Confidence** | | | | | | | | | | | | |
| **Sex*Area** | | | **N** | | **Mean** | | | **Grouping** | | | |  |
| Male A2 | | | 99 | | 0.288168 | | | A |  |  | |  |
| Female A2 | | | 124 | | 0.229727 | | |  | B |  | |  |
| Female A1 | | | 138 | | 0.209008 | | |  | B |  | |  |
| Male A1 | | | 31 | | 0.174722 | | |  |  | C | |  |
| Means that do not share a letter are significantly different. | | | | | | | | | | | |  |

| Worksheet 1 | | |  | |  | |  | |  | |  | |  |
| --- | --- | --- | --- | --- | --- | --- | --- | --- | --- | --- | --- | --- | --- |
| General Linear Model: High.Freq versus Sex, Area | | | | | | | | | | |  | |  |
| **Method** | | |  | |  | |  | |  | |  | |  |
| Factor coding | (-1, 0, +1) | |  | |  | |  | |  | |  | |  |
|  |  | |  | |  | |  | |  | |  | |  |
| Box-Cox transformation | | |  | |  | |  | |  | |  | |  |
| Rounded λ | -2 | |  | |  | |  | |  | |  | |  |
| Estimated λ | -1.55902 | |  | |  | |  | |  | |  | |  |
| 95% CI for λ | (-1.55952, -1.55852) | | | |  | |  | |  | |  | |  |
| **Factor Information** | | | | |  | |  | |  | |  | |  |
| **Factor** | **Type** | | **Levels** | | **Values** | |  | |  | |  | |  |
| Sex | Fixed | | 2 | | F, M | |  | |  | |  | |  |
| Area | Fixed | | 2 | | A1, A2 | |  | |  | |  | |  |
| **Analysis of Variance for Transformed Response** | | | | | | | | | | |  | |  |
| **Source** | **DF** | | **Adj SS** | | **Adj MS** | | **F-Value** | | **P-Value** | |  | |  |
| Sex | 1 | | 0 | | 0 | | 197.69 | | 0 | |  | |  |
| Area | 1 | | 0 | | 0 | | 387.1 | | 0 | |  | |  |
| Sex*Area | 1 | | 0 | | 0 | | 7.8 | | 0.005 | |  | |  |
| Error | 388 | | 0 | | 0 | |  | |  | |  | |  |
| Worksheet 1 | | | |  | |  | |  | |  | |  | |
| Comparisons for High.Freq | | | | | | | |  | |  | |  | |
| **Tukey Pairwise Comparisons: Sex** | | | | | | | |  | |  | |  | |
| **Grouping Information Using the Tukey Method and 95% Confidence** | | | | | | | | | | | | | |
| **Sex** | | **N** | | **Mean** | | **Grouping** | | | |  | |  | |
| F | | 262 | | 7928.26 | | A | |  | |  | |  | |
| M | | 130 | | 6445.24 | |  | | B | |  | |  | |
| Means that do not share a letter are significantly different. | | | | | | | | | | | |  | |
| **Tukey Pairwise Comparisons: Area** | | | | | | | |  | |  | |  | |
| **Grouping Information Using the Tukey Method and 95% Confidence** | | | | | | | | | | | | | |
| **Area** | | **N** | | **Mean** | | **Grouping** | | | |  | |  | |
| A2 | | 223 | | 8368.55 | | A | |  | |  | |  | |
| A1 | | 169 | | 6237.52 | |  | | B | |  | |  | |
| Means that do not share a letter are significantly different. | | | | | | | | | | | |  | |
| **Tukey Pairwise Comparisons: Sex*Area** | | | | | | | | | |  | |  | |
| **Grouping Information Using the Tukey Method and 95% Confidence** | | | | | | | | | | | | | |
| **Sex*Area** | | **N** | | **Mean** | | **Grouping** | | | | | | | |
| F A2 | | 124 | | 10321.7 | | A | |  | |  | |  | |
| M A2 | | 99 | | 7222.2 | |  | | B | |  | |  | |
| F A1 | | 138 | | 6676.8 | |  | |  | | C | |  | |
| M A1 | | 31 | | 5874.9 | |  | |  | |  | | D | |
| Means that do not share a letter are significantly different. | | | | | | | | | | | |  | |

| Worksheet 1 | | |  | |  | |  | |  | |  |  |
| --- | --- | --- | --- | --- | --- | --- | --- | --- | --- | --- | --- | --- |
| General Linear Model: Low.Freq versus Sex, Area | | | | | | | | | | |  |  |
| **Method** | | |  | |  | |  | |  | |  |  |
| Factor coding | (-1, 0, +1) | |  | |  | |  | |  | |  |  |
|  |  | |  | |  | |  | |  | |  |  |
| Box-Cox transformation | | |  | |  | |  | |  | |  |  |
| Rounded λ | 2 | |  | |  | |  | |  | |  |  |
| Estimated λ | 2.16347 | |  | |  | |  | |  | |  |  |
| 95% CI for λ | (1.40597, 2.91197) | | | |  | |  | |  | |  |  |
| **Factor Information** | | | | |  | |  | |  | |  |  |
| **Factor** | **Type** | | **Levels** | | **Values** | |  | |  | |  |  |
| Sex | Fixed | | 2 | | F, M | |  | |  | |  |  |
| Area | Fixed | | 2 | | A1, A2 | |  | |  | |  |  |
| **Analysis of Variance for Transformed Response** | | | | | | | | | | |  |  |
| **Source** | **DF** | | **Adj SS** | | **Adj MS** | | **F-Value** | | **P-Value** | |  |  |
| Sex | 1 | | 6.94E+11 | | 6.94E+11 | | 12.08 | | 0.001 | |  |  |
| Area | 1 | | 8.81E+12 | | 8.81E+12 | | 153.36 | | 0 | |  |  |
| Sex*Area | 1 | | 8.53E+12 | | 8.53E+12 | | 148.52 | | 0 | |  |  |
| Error | 388 | | 2.23E+13 | | 57443485230 | |  | |  | |  |  |
| Total | 391 | | 3.47E+13 | |  | |  | |  | |  |  |
| Worksheet 1 | | | |  | |  | |  | |  | |  |
| Comparisons for Low.Freq | | | | | | | |  | |  | |  |
| **Tukey Pairwise Comparisons: Sex** | | | | | | | |  | |  | |  |
| **Grouping Information Using the Tukey Method and 95% Confidence** | | | | | | | | | | | | |
| **Sex** | | **N** | | **Mean** | | **Grouping** | | | |  | |  |
| F | | 262 | | 1185.07 | | A | |  | |  | |  |
| M | | 130 | | 1142.09 | |  | | B | |  | |  |
| Means that do not share a letter are significantly different. | | | | | | | | | | | |  |
| **Tukey Pairwise Comparisons: Area** | | | | | | | |  | |  | |  |
| **Grouping Information Using the Tukey Method and 95% Confidence** | | | | | | | | | | | | |
| **Area** | | **N** | | **Mean** | | **Grouping** | | | |  | |  |
| A2 | | 223 | | 1237.97 | | A | |  | |  | |  |
| A1 | | 169 | | 1084.52 | |  | | B | |  | |  |
| Means that do not share a letter are significantly different. | | | | | | | | | | | |  |
| **Tukey Pairwise Comparisons: Sex*Area** | | | | | | | | | |  | |  |
| **Grouping Information Using the Tukey Method and 95% Confidence** | | | | | | | | | | | | |
| **Sex*Area** | | **N** | | **Mean** | | **Grouping** | | | | | |  |
| M A2 | | 99 | | 1287.61 | | A | |  | |  | |  |
| F A2 | | 124 | | 1186.27 | |  | | B | |  | |  |
| F A1 | | 138 | | 1183.87 | |  | | B | |  | |  |
| M A1 | | 31 | | 975.1 | |  | |  | | C | |  |
| Means that do not share a letter are significantly different. | | | | | | | | | | | |  |

| Worksheet 1 | | |  | |  | |  | |  | |  |  |
| --- | --- | --- | --- | --- | --- | --- | --- | --- | --- | --- | --- | --- |
| General Linear Model: Peak.Freq versus Sex, Area | | | | | | | | | | |  |  |
| **Method** | | |  | |  | |  | |  | |  |  |
| Factor coding | (-1, 0, +1) | |  | |  | |  | |  | |  |  |
|  |  | |  | |  | |  | |  | |  |  |
| Box-Cox transformation | | |  | |  | |  | |  | |  |  |
| Rounded λ | 0 | |  | |  | |  | |  | |  |  |
| Estimated λ | -0.0428864 | |  | |  | |  | |  | |  |  |
| 95% CI for λ | (-0.256386, 0.169614) | | | |  | |  | |  | |  |  |
| **Factor Information** | | | | |  | |  | |  | |  |  |
| **Factor** | **Type** | | **Levels** | | **Values** | |  | |  | |  |  |
| Sex | Fixed | | 2 | | F, M | |  | |  | |  |  |
| Area | Fixed | | 2 | | A1, A2 | |  | |  | |  |  |
| **Analysis of Variance for Transformed Response** | | | | | | | | | | |  |  |
| **Source** | **DF** | | **Adj SS** | | **Adj MS** | | **F-Value** | | **P-Value** | |  |  |
| Sex | 1 | | 0.051 | | 0.05098 | | 0.64 | | 0.426 | |  |  |
| Area | 1 | | 1.7083 | | 1.7083 | | 21.32 | | 0 | |  |  |
| Sex*Area | 1 | | 0.0286 | | 0.02858 | | 0.36 | | 0.551 | |  |  |
| Error | 388 | | 31.0846 | | 0.08012 | |  | |  | |  |  |
| Total | 391 | | 33.5623 | |  | |  | |  | |  |  |
| Worksheet 1 | | | |  | |  | |  | |  | |  |
| Comparisons for Peak.Freq | | | | | | | |  | |  | |  |
| **Tukey Pairwise Comparisons: Sex** | | | | | | | |  | |  | |  |
| **Grouping Information Using the Tukey Method and 95% Confidence** | | | | | | | | | | | | |
| **Sex** | | **N** | | **Mean** | | **Grouping** | |  | |  | |  |
| M | | 130 | | 2439 | | A | |  | |  | |  |
| F | | 262 | | 2374 | | A | |  | |  | |  |
| Means that do not share a letter are significantly different. | | | | | | | | | | | |  |
| **Tukey Pairwise Comparisons: Area** | | | | | | | |  | |  | |  |
| **Grouping Information Using the Tukey Method and 95% Confidence** | | | | | | | | | | | | |
| **Area** | | **N** | | **Mean** | | **Grouping** | | | |  | |  |
| A2 | | 223 | | 2603 | | A | |  | |  | |  |
| A1 | | 169 | | 2225 | |  | | B | |  | |  |
| Means that do not share a letter are significantly different. | | | | | | | | | | | |  |
| **Tukey Pairwise Comparisons: Sex*Area** | | | | | | | | | |  | |  |
| **Grouping Information Using the Tukey Method and 95% Confidence** | | | | | | | | | | | | |
| **Sex*Area** | | **N** | | **Mean** | | **Grouping** | | | | | |  |
| M A2 | | 99 | | 2665 | | A | |  | |  | |  |
| F A2 | | 124 | | 2542 | | A | | B | |  | |  |
| M A1 | | 31 | | 2232 | |  | | B | | C | |  |
| F A1 | | 138 | | 2217 | |  | |  | | C | |  |
| Means that do not share a letter are significantly different. | | | | | | | | | | | |  |
